# Supplementary material for: What motivates consumers to buy organic foods? Results of an empirical study in the United States
Source: PLoS One. 2021 Sep 10;16(9):e0257288. doi: 10.1371/journal.pone.0257288 (PMC8432837; doi:10.1371/journal.pone.0257288)
Supplement: S1 Appendix — (DOCX) [file pone.0257288.s002.docx]

**S1 Appendix: Survey Questionnaire**

Q1. Have you participated in any survey related to organic food during Spring 2018?

Yes (1) No (2)

Skip To: End of Survey If Q1 = Yes

Q2. Do you purchase organic food products?

Definitely yes (1) Probably yes (2) Might or might not (3)

Probably not (4) Definitely not (5)

Skip To: Q3 If Q2 = Probably not

Skip To: Q3 If Q2 = Definitely not

Skip To: Q3 If Q2 = Might or might not

Q3. Please indicate the reason for choosing organic foods? Choose as many as apply.

Healthy Contents (1) Pesticide-Free (2) Freshness (3)

Environmentally Friendly (4) Tastes Better (5) To try something new (6)

Trendy & Fashionable (7)

***Please answer the following statements.***

Q4. I am concerned about the type and amount of nutrition in the food that I consume daily?

Strongly agree (1) Somewhat agree (2) Neither agree, nor disagree (3)

Somewhat disagree (4) Strongly disagree (5)

Q5. Organic foods are good for my health.

Strongly agree (1) Somewhat agree (2) Neither agree, nor disagree (3)

Somewhat disagree (4) Strongly disagree (5)

Q6. I am not willing to buy a lot of products, to eat as healthy as possible.

Strongly agree (1) Somewhat agree (2) Neither agree, nor disagree (3)

Somewhat disagree (4) Strongly disagree (5)

Q7. I want to know if the product is organic or non-organic before I make a purchase.

Strongly agree (1) Somewhat agree (2) Neither agree, nor disagree (3)

Somewhat disagree (4) Strongly disagree (5)

Q8. I know how organic foods are produced.

Strongly agree (1) Somewhat agree (2) Neither agree, nor disagree (3)

Somewhat disagree (4) Strongly disagree (5)

Q9. I believe that organic foods are safer for consumption.

Strongly agree (1) Somewhat agree (2) Neither agree, nor disagree (3)

Somewhat disagree (4) Strongly disagree (5)

Q10. My friends and family consume organic food products.

Strongly agree (1) Somewhat agree (2) Neither agree nor disagree (3)

Somewhat disagree (4) Strongly disagree (5)

Q11. My loved ones expect me to purchase more organic food products for them.

Strongly agree (1) Somewhat agree (2) Neither agree, nor disagree (3)

Somewhat disagree (4) Strongly disagree (5)

Q12. Many people persuade me that I should buy organic food products for better lives.

Strongly agree (1) Somewhat agree (2) Neither agree, nor disagree (3)

Somewhat disagree (4) Strongly disagree (5)

Q13. Organic food products are expensive.

Strongly agree (1) Somewhat agree (2) Neither agree, nor disagree (3)

Somewhat disagree (4) Strongly disagree (5)

Q14. The price of organic food products is per their benefits.

Strongly agree (1) Somewhat agree (2) Neither agree, nor disagree (3)

Somewhat disagree (4) Strongly disagree (5)

Q15. Organic food products are readily available in the market.

Strongly agree (1) Somewhat agree (2) Neither agree, nor disagree (3)

Somewhat disagree (4) Strongly disagree (5)

Q16. I can buy organic food products online.

Strongly agree (1) Somewhat agree (2) Neither agree, nor disagree (3)

Somewhat disagree (4) Strongly disagree (5)

Q17. I believe organic foods are useful in meeting my nutritional needs.

Extremely useful (1) Very useful (2) Moderately useful (3)

Slightly useful (4) Not at all useful (5)

Q18. Organic food products have higher quality than conventional products.

Strongly agree (1) Somewhat agree (2) Neither agree, nor disagree (3)

Somewhat disagree (4) Strongly disagree (5)

Q19. I am convinced that the consumption of organic foods is a reasonable action.

Strongly agree (1) Somewhat agree (2) Neither agree, nor disagree (3)

Somewhat disagree (4) Strongly disagree (5)

Q20. I intend to consume organic food products in the future.

Strongly agree (1) Somewhat agree (2) Neither agree, nor disagree (3)

Somewhat disagree (4) Strongly disagree (5)

Q21. I always intend to purchase organic food products.

Strongly agree (1) Somewhat agree (2) Neither agree, nor disagree (3)

Somewhat disagree (4) Strongly disagree (5)

Q22. I buy organic food products regularly.

Strongly agree (1) Somewhat agree (2) Neither agree, nor disagree (3)

Somewhat disagree (4) Strongly disagree (5)

Q23. I still buy organic food products even if the conventional alternatives are on sale.

Strongly agree (1) Somewhat agree (2) Neither agree, nor disagree (3)

Somewhat disagree (4) Strongly disagree (5)

Q24. I never mind paying a premium price for organic food products.

Strongly agree (1) Somewhat agree (2) Neither agree, nor disagree (3) Somewhat disagree (4) Strongly disagree (5)

***Please answer a few questions about yourself.***

Q25. Your Gender? Male (1) Female (2)

Q26. Your Age?

18 – 30 years (1) 31 – 40 years (2) 41 to 50 years (3)

51 – 60 years (4) Above 60 years (5)

Q27. Your educational qualification.

High School (1) Undergraduate (2)

Graduate (3) Post Graduate (Ph.D. etc.) (4)

Q28. How many people are in your family (including you).

1 – 2 (1) 3 – 4 (2) 5 or more (3)

Q29. Please indicate your family's annual income.

Less than 40,000 (1) 40,001 to 60,000 (2) 60,001 to 80,000 (3)

80,001 to 100,000 (4) Above 100,001 (5)

Q30. Please indicate your occupation.

Student (1) Work Full-time (2) Self-Employed (3) Retired (4)

Q31. Please indicate the reason why you are not purchasing organic food products?

________________________________________________________________

Q32. What factor can motivate you to purchase an organic food products? Please indicate

________________________________________________________________

Skip To: End of Survey If Q4 Is Contains

End of Block: Default Question Block
