# Supplementary material for: What motivates consumers to buy organic foods? Results of an empirical study in the United States
Source: PLoS One. 2021 Sep 10;16(9):e0257288. doi: 10.1371/journal.pone.0257288 (PMC8432837; doi:10.1371/journal.pone.0257288)
Supplement: Attachment — Submitted filename: Response to Reviewers.docx [file pone.0257288.s003.docx]

**Response to Reviewers**

We thank the editor and the reviewers for their very thoughtful comments on our paper. All the suggestions and queries of reviewers have been considered carefully. We have responded to each comment and made appropriate changes to the manuscript. Reviewers and editor’s comments are in bold, authors’ responses are in plain text.

**Reviewer#1**

**1) I am uncomfortable with the title where the focus seems to be on consumers from Mid-western US. I understand that the authors may be located in the area and it is acceptable to focus in that region. However, given that your title is specifically about Midwest consumers, there needs to be a detailed section in the Literature review on why you chose consumers from that area. Any stats on mid-west consumers' spending on organic food? Why are they important compared to the rest of US consumers. If you cannot justify this, then I am uncomfortable with the entire study and you should label the data as convenience sample.**

We agree with the reviewer's comment on the title. We changed to title and removed the word 'mid-western. Since there is no difference with the rest of the US consumers, it isn't easy to justify this. The data details are changed to a convenience sample.

**2) The limitations of this study should include that the data was collected by means of a snowball sampling technique.**

It is added to the limitations.

**3) With respect to the survey questions, did you disqualify respondents if they did not "purchase organic products" (Q2)?**

No. Respondents who did not purchase are also considered for the survey. We are interested in knowing what factors might influence them to purchase in the future.

**4) For Q2 above, you should have indicated a time frame on when they purchased organic products in the past? Why did you generalize as "organic products" and not "organic food"?**

The reason for not adding a time frame is to avoid 'recall error.' It might be difficult for the respondent to remember the time frame of purchase.

Regarding the “organic products”, we are extremely sorry that old version of questionnaire was appended. Intially, we were interested in studying the consumer buying behaviour toward organic products. But, during the pilot survey we learned that “organic products” would be a too broad term which includes clothing and personal care items, and consumers may view or behave differently for different items such as food, clothing and personal care. Therefore, in the final survey, we used the term ‘*organic food products’*. We thank the reviewer for highlighting it. The final version of questionnaire is appended in the revised manuscript.

**5) The first line under Conclusion indicates that the study tested the research model on US consumers. You cannot generalize your conclusions to all of US consumers if you are just focusing on Mid-west consumers.**

Noted, and the changes are made in the document by removing the word 'mid-western consumers.'

**Reviewer#2**

Thank you for the feedback.

**Response to editor**

1) **Please revise the statistical methods employed in this study. For instance, the probability value is never equal to zero. More accurately, it is always above zero.**

We agree with the editor that probability (in this case, p-value) is always above zero. Using SPSS software, in our tables, p-values are rounded off to three decimal places. Using built-in rounding rules, it was rounded down and reported as .000. The value reported as **.000** is not absolute zero or exctally zero. The probability (p-value) is very small, however, there are still some chances of occurance of the event. Just to bring clarity, we have provided notes to the table in the revised manuscript. Thanks for highlighting it.

2) **COVID perceptions effects on adopting healthy food habits are not investigated. Thus, this must be addressed as a research limitation/implication by citing [1, 2]. Also, parental attitudes need to be highlighted for future research [1, 3].**

Noted. We have incorporated the suggestions in the revised manuscript.
